# Design Principle and Proofing of a New Smart Textile Material That Acts as a Sensor for Immobility in Severe Bed-Confined Patients

**DOI:** 10.3390/s23052573

**Published:** 2023-02-25

**Authors:** Bogdan Florin Iliescu, Vlad Niki Mancasi, Ionut Dumitru Ilie, Iulian Mancasi, Bogdan Costachescu, Daniel Ilie Rotariu

**Affiliations:** 1 Department of Neurosurgery, “Gr T Popa” University of Medicine and Pharmacy Iasi, 700115 Iasi, Romania; 2 School of Industrial Design and Business Management, Gh. Asachi University of Iasi, 700050 Iasi, Romania; 3 Majutex Inc., 707035 Iasi, Romania

**Keywords:** smart textile, immobility, sensor, conductive

## Abstract

The immobility of patients confined to continuous bed rest continues to raise a couple of very serious challenges for modern medicine. In particular, the overlooking of sudden onset immobility (as in acute stroke) and the delay in addressing the underlying conditions are of utmost importance for the patient and, in the long term, for the medical and social systems. This paper describes the design principles and concrete implementation of a new smart textile material that can form the substrate of intensive care bedding, that acts as a mobility/immobility sensor in itself. The textile sheet acts as a multi-point pressure-sensitive surface that sends continuous capacitance readings through a connector box to a computer running a dedicated software. The design of the capacitance circuit ensures enough individual points to provide an accurate description of the overlying shape and weight. We describe the textile composition and circuit design as well as the preliminary data collected during testing to demonstrate the validity of the complete solution. These results suggest that the smart textile sheet is a very sensitive pressure sensor and can provide continuous discriminatory information to allow for the very sensitive, real-time detection of immobility.

## 1. Introduction

Patient immobility represents a very serious medical condition that is still largely unaddressed and results in at least two very serious implications. First, it has been linked to a number of serious and costly hospital complications, including pressure ulcers, pneumonia, falls, DVT/PE, and muscle deconditioning. Each year, billions of unreimbursed healthcare dollars are spent treating complications related to patient immobility. Secondly, the sudden onset of immobility, partial or general, in a previously mobile patient usually indicates the onset of a very serious neurological condition, more often than not life-threatening unless treated in the shortest time possible from onset. For example, between 2.2% and 17% of all strokes have symptom onset during hospitalization in a patient originally admitted for another diagnosis or procedure [1,2,3,4]. These in-hospital strokes represent a unique population with different risk factors, more mimics, and substantially worsened outcomes compared to community-onset strokes [2,5]. The lifetime direct and indirect costs for these in-hospital strokes are approximately 4.9 billion to 10.5 billion dollars [6,7]. Given that the comorbidity of in-hospital strokes is higher than in community strokes, these are likely to be conservative estimates.

Another extremely serious complication comes in the form of pressure ulcers. Pressure ulcers are chronic wounds with physiologically impaired healing [8,9]. An estimated 15% of acute care patients have pressure ulcers [10], and pressure ulcer incidence has increased by 63% in recent years [11]. Pressure ulcers are an epidemic among bed-bound populations, with a reported prevalence as high as 26% among hospitalized patients [12], 43% among those in nursing homes, and 39% among patients with spinal cord injuries [13]. The morbidities associated with pressure ulcers represent a considerable healthcare problem, particularly when healing does not occur [14]. Approximately 50% of stage II [15] and 95% of stage III and IV pressure ulcers do not heal within 8 weeks [16]. Patients with pressure ulcers usually demonstrate significantly impaired physical and social function, self-care, and mobility [17]. Common associated morbidities include pain, depression, local infection, anemia, osteomyelitis, and sepsis [14,18,19,20,21]. In addition, patients with pressure ulcers often require either long-term hospitalization or frequent hospital admissions. The presence or development of a pressure ulcer can increase the length of a patient’s hospital stay by an average of 10.8 days [22]. These extended hospitalizations are associated with higher costs and increased incidence of nosocomial infection and/or other complications [23]. In one study [24], the hospital cost averaged USD 127,185 over a maximum of 29 months. The cost for a hospital-acquired pressure ulcer patient averaged USD 129,248 during one hospital stay, whereas the cost for a community-acquired pressure ulcer patient averaged USD 124,327 for stage 4 ulcers.

Medical textiles have for a while been the target of an intensive research effort in various fields as a valuable resource to improve the level of care and to introduce smart sensors in healthcare. This has resulted in the investigation and production of wearable textile-based systems for healthcare and compounds for Ambient Assisted Living (AAL) environments. The first attempts to manufacture biomonitoring clothing started over a decade ago, and there have been a great number of reports published on investigations of separate compound and complex systems’ development. Smart materials find applications in hospital textiles and clothing for medical personnel. Additional functionality of such textiles can be obtained by different approaches according to specific applications. For this type of medical textile though, most solutions are brought by functional textiles. For hospital textiles, those commonly used are textile materials with antimicrobial and antibacterial properties or low-friction coating. Clothing for medical personnel is also made of functional textiles that ensure efficient moisture transport and biological protection.

Nevertheless, conductive textile materials are more often used in the manufacturing of heating textiles that find applications in blankets for operating rooms. Beyond that, conductive textile materials can be an asset in the improvement of distance communication between medical personnel and patients through wearable technologies integrated into clothing. Smart textiles also offer a solution for decubitus prophylaxis and related health disorders that are a significant problem in the hospital environment. At present, there are a number of developments that assist in managing these problems through innovative and smart textile solutions. Namely, those that can be implemented by stimulating blood flow in sensitive areas via textile-based sensors and systems, or by optimizing and controlling moisture management via textile sensors [25]. The first known attempts in textile sensor development were reported over a decade ago within such research projects as Wearable MotherboardTM by Georgia Tech, and such EU projects as Wealthy and My Heart (IWearable Motherboards, 2013) [26,27,28]. At present, there are a great variety of textile sensors and complete systems for the assessment of physiological and biochemical processes. As well as biomonitoring functions, smart sensors can assist in such prophylactic and therapeutic arrangements as muscle electrical stimulation and posture monitoring [29,30,31,32].

Thus far, the problem of patient bed mobility monitoring has been addressed through the integration of various sensor technologies within normal medical textiles. We propose a novel solution that makes the textile itself smart. From breathing monitoring to mobility defined for every segment of the body, the smart sheet provides reliable, continuous, low energy patient monitoring and stays in touch with the caregivers through enhanced communication channels. The computer-based and mobile applications ensure a straight and accessible way of monitoring and manages the entire liaison between the patient and those who nurse them.

## 2. Materials and Methods

### 2.1. Textile Material Design and Composition

The textile material is produced with a woven structure, having 100% polyester threads in the composition in the warp and weft, the rips of the warp having a mild hydrophobic character. A material with a fineness of 330 dtex and a weight of 180 g/m^2^ is obtained.

**Figure 1:**
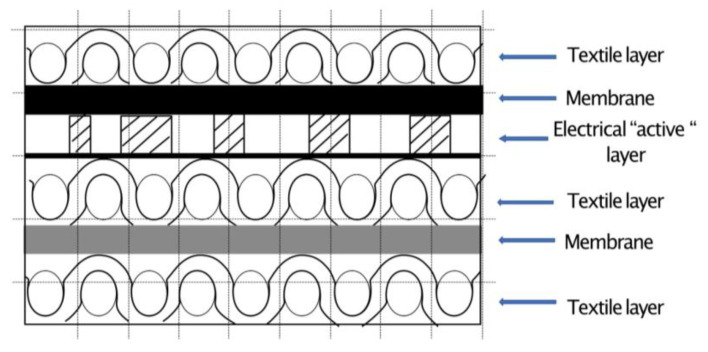
Schematics of the textile material structure in coronal section.

The most important type of polyester is polyethylene terephthalate (PET), commonly referred to simply as polyester. This is the product of condensation reaction between ethylene diglycol and terephthalic acid. The macromolecular chain of polyethylene terephthalate is linear, devoid of ramifications, benzene nuclei are arranged in the same plane, allowing the realization of numerous intermolecular bonds and implicitly obtaining an ordered and compact structure. The intermolecular bonds are of Van der Waals type. These are weaker compared to hydrogen bonds, but the compact packaging creates numerous connections that are positively reflected in the behaviour of the fibers at mechanical demands. The lack of hydrophilic groups as well as the compact structure imprints the fibers a hydrophobic character, a very good stability in wet environment, and a high charging capacity with electrostatic charges. The polyester yarns used in this experiment are all filament with variations such as: textured and flat (167 dtex drawn textured yarn in warp with 234 yarns/10 cm and 333 dtex drawn textured yarn with 160 yarns/10 cm). One key difference between polyester and polyamide fibers is that polyester fibers are far more resistant to sunlight. Polyester fibers are also resistant to the action of dilute acids, alkalis and organic solvents but can be badly damaged at high concentrations (Figure 1).

The other important aspect of the final product is the structure of the fabric. Having as the strongest consideration the final use of the textile material, we chose a structure that avoids big floats and focuses on strength, rather than design features (drape, shine, covering ability, etc.). More important aspects were taken into account when designing the textile structure: isotropy (has more to do with the fabric’s stability on all directions) and wettability (plays a huge roll when applying the paste on the fabric). The structure chosen for this application is plain weave due to its high tensile work (because of more floating points).

As well as having the suitable yarn and the appropriate structure, special attention was dedicated to the finishing of the fabric. The first step in finishing is the desizing of the fabric, where oligomers and other fats and oils are removed from the surface of the yarn. This allows for a better paste application on the yarn, or we would be faced with reduced fastness. The second step in finishing was the impregnation of the fabric with a very light (<20 g/L) oleophobic solution, through padding. The pick-up is approximately 50%. This step was necessary to prevent the conductive paste from sinking too much in the fabric, as the paste needs to be uniformly distributed on top of the fabric, rather than between the yarns. This is a strict condition for not influencing the electrical resistance of the electrical circuit. After having the fabric impregnated with the light oleophobic solution, the next step was to heat set the fabric at approx. 195 degrees Celsius for approx. 60 **s** in order to reduce internal tensions, which ultimately leads to a very low shrinkage factor (<0.5%)

**Figure 2:**
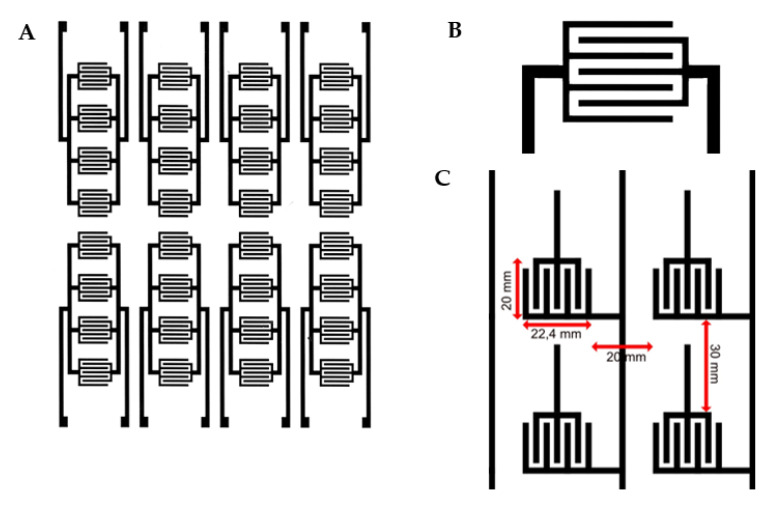
The pattern printed on the surface of the textile polyester base. (**A**) Macro view as it appears on the material. (**B**) Detail of an individual active “cell”. (**C**) Details of sensor size and sensors’ spacing within the scheme. Each sensor works as a capacitor.

On one surface of the material, the electrical circuit is printed through printing screen procedure using a water-based conductive paste with carbon nanotubes. The conductive paste is responsible for the electrical conductivity and resistance of the printed circuit with values between 0.6 and 2 kOhm/mL. The resulting printed circuit contains 5600 active cells with independent potential per square meter of material, each cell having a surface of 1 × 2 cm. An individual cell consists of a “U”-shaped anode and a cathode in the middle (Figure 2A). The cells are continuous on the entire printed surface of the textile material, as can be seen in Figure 2B.

The sensors are divided in 3 layers on top of one another, consisting of: first layer of conductive paste, second layer of dielectric paste, and third layer again of conductive paste. These three layers are applied on the fabric’s surface via screen printing technology, using three different screens (see attached documents). All screens are of the same type, meaning mesh 40 HX. This stands for 30% open surface, with hole diameter of 345 microns. This type of screen makes it the most suitable for this application, taking into consideration that the applied conductive paste has a viscosity of approx. 65 dPas (high viscosity, with high risk of clogging up the screen). This conductive paste is an aqueous dispersion based on polyurethane and carbon. Carbon facilitates the electric charge transport, whereas the polyurethane is there for improving the mechanical properties of the paste (flexibility, resistance to abrasion, etc.). The dielectric paste, which is placed in the middle of the conductive paste layers, is based on polyurethane, for avoiding a short circuit between first and third layers. The polyurethane polymer found in the composition of the printing paste ensures a mechanical resistance of the printed circuit, which guarantees a consistent electrical behavior over time under repeated manipulations (twisting, traction, compression). It also provides the hydrophobic property, which guarantees the maintenance of the electric properties regardless of the environment in which it is used.

Considering the pattern length, which is also the screen length (64 cm), is significantly smaller than that of a mattress cover, it is obvious that such a product will have more circuit patterns on it. This means that the data collection will be performed through more patterns (3). In the last step, the textile material printed with the described electric circuit is laminated with hydrophobic membrane on both surfaces over which textile layers are applied made of 100% polyester for circuit protection. This would be conducted by means of hot-melt adhesive membrane of approx. 50 microns, with high water and heat fastness.

### 2.2. Connector Box Development and Integration

**Figure 3:**
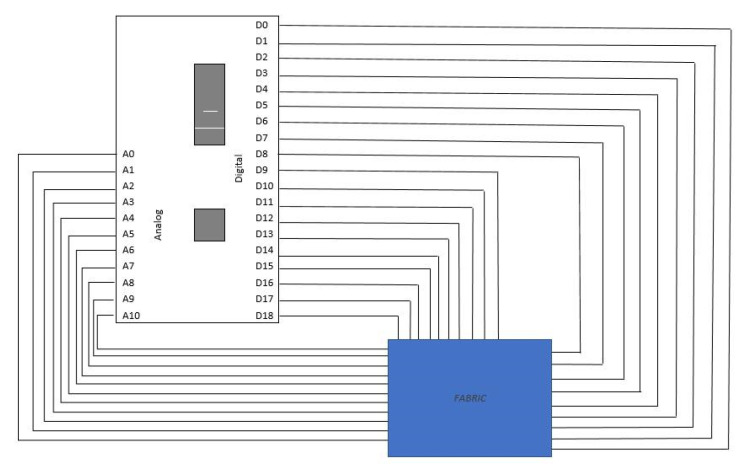
The basic electrical schematic of the connector box.

In charge of reading the signals generated within the textile printed circuit, we placed an Arduino electronic development board. The board uses physical connections with the outputs of the printed circuit to collect and integrate the potentials from the entire textile surface. The “smart fabric” is connected to three controller boxes that collect the signal from the fabric layers. The signal is splatted in two sides: 11 analogic pins and 19 digital pins (Figure 3). Each conductive line is connected to a prototyping board with thin wire that has Ag insertion and soldered to controller box. The controller box receives, records, and converts the signal into valuable data such as “body map”. In fact, this controller converts the signal received to numbers, values, and graphics. Signal interpretation is performed using a source code uploaded to the controller.

In the second step, the board transforms the raw signals into digital signals and sends them via USB to the computing unit. The electronic board collects the potentials from a matrix of 66 cathode connections and one anodic connection. Each connection collects the potentials generated by 73 individual cells. The information collected from the electronic board is interpreted by a dedicated software that displays them graphically in real time.

### 2.3. Software Development

**Figure 4:**
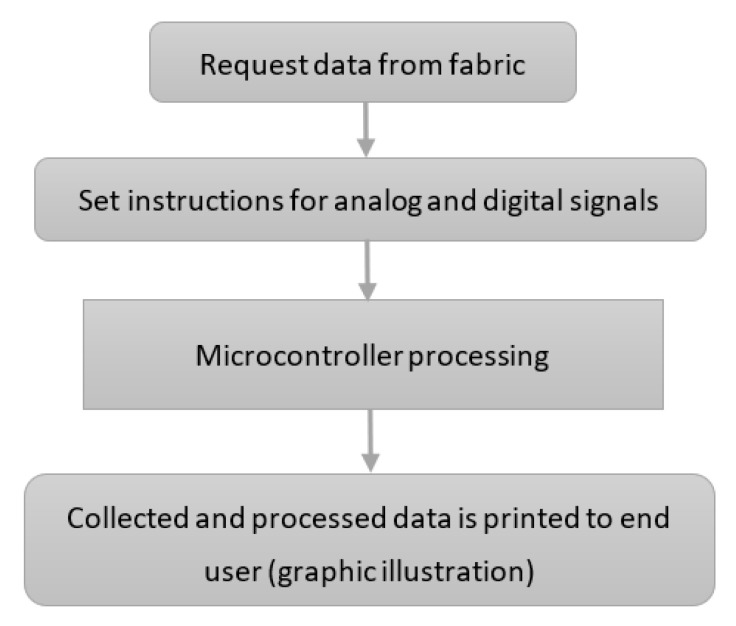
Flow diagram of the software processing the signals from the material.

We developed a source code that collects the information from the fabric. The code is uploaded on the controller box. The basic code flow is presented in Figure 4.

Data are requested from the fabric, the code reads the analogue and digital pins from the controller box. Different sets of instructions process the analogue and the digital signals, respectively. After their processing, the collected data are sent to a graphical display and to a file that records all processed data. As the example clearly shows, the signal is collected in a “row” and “column” fashion for better accuracy. We mention that this part of the prototype is still under improvement work in order to make the visual representation as intuitive and easily interpretable as possible.

## 3. Results—Preliminary Test Data

We created a smart textile that is capable of transforming pressure applied discriminatorily on its surface into electrical signals. The signals are recorded and processed by an external electronic board that send the digital information to a dedicated software, which, in turn, plots it visually. The material uses a polyester textile base on which a specific pattern of conductive dye is printed. The resulting circuit is then linked to the pins of the external electronic board.

**Figure 5:**
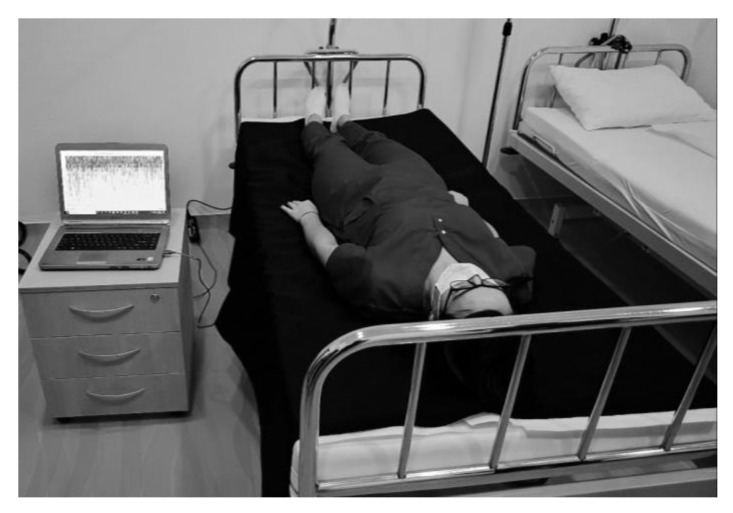
Preliminary real-life test setup.

In order to evaluate the applicability of our smart sheet sensor in a real-life situation we devised a test setup comprising of a hospital bed covered with our smart sheet and running monitoring software (Figure 5. A volunteer lay on the bed and alternated between periods of immobility and normal movements, including positional changes. The analysis of the real-life behavior of the smart sheet gave important insights into its function as a medical device and upgrades that could increase its practical value. The analysis showed that the smart sheet successfully reached the main goals that it was designed to achieve.

The entire surface of the sheet behaved similarly to a discriminatory pressure-sensitive surface. All sense points were active and the application could discriminate each of them. This aspect is essential in order to create a sensor with the sensibility required, especially by the medical application. The adjacent sense points were easily readable and plotted in the graphical display. The differences were clearly visible and discernable on the display, the entire construct showing a good sensitivity, fundamental for clinical applications. Signal transmission and processing were performed consistently in real time. The graphical plot followed closely and accurately the motion of the patient on the surface of the sheet. The pattern of values made it possible to attain an accurate reproduction of the pressure put on the surface with every motion, even if the patient was not in the visual range. The smart sheet proved to have a very good temporal resolution alongside its excellent spatial resolution.

During the whole duration of the testing, there was no artificial decrease in the intensity of the signals nor any other malfunctions in the system. No external factors interfered with the good functioning and monitoring provided by the smart sheet. The sheet proved to be indifferent to mechanical factors, temperature, bending, and stretching (during the different motions of the patient on its surface). It proved to have a stable reliability in time consistent with the desired behavior. The sheet has good ergonomics. Its surface did not incommode the patients during the entire duration of the tests. It was easy to place on the beds and easy to remove. Its physical properties did not intervene in any way with the behavior of the subjects (they did not move as a result of sores or other sheet-related issues) or of the application.

Although not yet tested for its physical properties (the regular properties of a polyester textile can be translated), during the whole testing process, the material did not change behavior. We cannot make any assumptions on its behavior after washing or other chemical treatment; however, its resistance to tear and wear seem appropriate for its function.

## 4. Discussion

The challenge of the early detection of a newly installed motor deficit and the difficulties in evaluating and managing long periods of bed confinement, make mobility monitoring a stringent problem to solve. Although solutions (anti-bedsore mattresses for example) have been proposed to reduce some of the prolonged bed confinement on patients, there are situations that are difficult to address and that account for numerous aggravations during a hospital stay. One simple and most common scenario is that of a newly developed acute stroke that is, in the vast majority of the cases, discovered beyond the useful early therapeutic window.

We propose a novel solution for real-time, sensitive, and reliable monitoring of bed-confined patients. Although the newly designed smart textile sheet sensor proved its capability in monitoring the mobility of a person under bed rest conditions, there are still further improvements and upgrades that can substantially increase the usability and clinical utility of the smart sheet.

A dynamic display, in real time, of the pressure map in 2D or 3D with a color code can show a real contour of the human body or can strictly reproduce the different levels of pressure in the different zones of the sheet. Medical specialists present at the test pointed out that the present display of the data collected from the sheet is unintuitive and impractical to use in hospital conditions. The position and pressure points of the patient’s body have to be clearly and unequivocally displayed in order to be easily interpreted from one look.

For an “alarms” setup, the application must monitor the dynamics of the contacts between the sheet and the “patient” and must follow long periods (≥30 min) of general or partial immobility (hemi-body, limb). When they appear, they must trigger the software to generate an alarm with both video and audio expressions and possibly send it by SMS/intranet/internet. The second type of alarm should occur when the dynamics of pressure are completely chaotic or repetitive (suggestive of a seizure), and in this case, the system generates an alarm with video and audio components and the possibility of remote sending. Medical personnel suggested that the alarm function could fill-in for the lack of observation of the human caregivers and is the best way to reduce the response time.

Quantification of the activation time and activation intensity of specified pressure zones could be necessary. When selecting an area of interest (sacral area or heel, for example), the application must display the total activation time and activation intensity. In this way, the decubitus ulcerations can be prevented more easily. The real-life test showed that the temporal and spatial resolution of the data collection from the smart sheet is appropriate. However, further improvement in correcting the delay from fact to display is a welcome upgrade to the current setup.

Further improvement in signal processing algorithms at the level of the connector box is necessary. On the strictly technical side, the way signals come from the surface of the smart sheet are interpreted first by the connector box. Improvements in the way the software later displays the data start at the level of the connector box. Hardware and machine coding changes might be necessary in order to accommodate the various display options. Software signal interpretation and plotting algorithms are one of the key aspects of the utility of the application. In order to improve the usability for the target groups (2D/3D body map vs. surface plotting with pressure color code map), these algorithms have to be changed and further adjustments made in order to meet the usability criteria.

Further improvement is necessary in the software regarding the way it monitors and records certain defined areas and pressure points in order to compare their values over time with the defined patterns of activation. This property is fundamental for the alarms’ definition and subsequent behavior of the application in the real, clinical environment. The development of a structured bug report system and bug solving has to be put in place. Clinical use implies that any malfunction or error that appears during its usage has to be solved in the shortest time possible. For this to be doable, a straight forward system of report and fix has to be active and efficient at the moment of implementation.

## 5. Conclusions

We present a novel smart textile material that can act as a very sensitive mobility detection sensor in itself and through dedicated software can act as a real-time immobility alert system. The smart sheet sensor is both comfortable and easy to use, and it is consistent during long periods of usage. Although it can be argued that there are improvements that can be made both to its components as well as usability, it can be a life-saving or brain-saving addition to ICU or neurology units as it can provide a very early alert of sudden onset immobility for bed-confined patients.

## 6. Patents

Pending patent No a 2021 00454/30.07.2021.

## Data Availability

No new data were created or analyzed in this study. Data sharing is not applicable to this article.
